# Investigation of Melioidosis Using Blood Culture and Indirect Hemagglutination Assay Serology among Patients with Fever, Northern Tanzania

**DOI:** 10.4269/ajtmh.20-0160

**Published:** 2020-09-28

**Authors:** Michael J. Maze, Mindy Glass Elrod, Holly M. Biggs, John Bonnewell, Manuela Carugati, Alex R. Hoffmaster, Bingileki F. Lwezaula, Deng B. Madut, Venance P. Maro, Blandina T. Mmbaga, Anne B. Morrissey, Wilbrod Saganda, Philoteus Sakasaka, Matthew P. Rubach, John A. Crump

**Affiliations:** 1 Department of Medicine, University of Otago, Christchurch, New Zealand;; 2 Centre for International Health, University of Otago, Dunedin, New Zealand;; 3 Kilimanjaro Christian Medical Centre, Moshi, Tanzania;; 4 Bacterial Special Pathogens Branch, US Centers for Disease Control, Atlanta, Georgia;; 5 Duke Global Health Institute, Duke University, Durham, North Carolina;; 6 Division of Infectious Diseases and International Health, Department of Medicine, Duke University Health System, Durham, North Carolina;; 7 Mawenzi Regional Referral Hospital, Moshi, Tanzania;; 8 Programme in Emerging Infectious Diseases, Duke-National University of Singapore, Singapore, Singapore;; 9 Kilimanjaro Clinical Research Institute, Moshi, Tanzania;; 10 Kilimanjaro Christian Medical University College, Tumaini University, Moshi, Tanzania

## Abstract

Prediction models indicate that melioidosis may be common in parts of East Africa, but there are few empiric data. We evaluated the prevalence of melioidosis among patients presenting with fever to hospitals in Tanzania. Patients with fever were enrolled at two referral hospitals in Moshi, Tanzania, during 2007–2008, 2012–2014, and 2016–2019. Blood was collected from participants for aerobic culture. Bloodstream isolates were identified by conventional biochemical methods. Non–glucose-fermenting Gram-negative bacilli were further tested using a *Burkholderia pseudomallei* latex agglutination assay. Also, we performed *B. pseudomallei* indirect hemagglutination assay (IHA) serology on serum samples from participants enrolled from 2012 to 2014 and considered at high epidemiologic risk of melioidosis on the basis of admission within 30 days of rainfall. We defined confirmed melioidosis as isolation of *B. pseudomallei* from blood culture, probable melioidosis as a ≥ 4-fold rise in antibody titers between acute and convalescent sera, and seropositivity as a single antibody titer ≥ 40. We enrolled 3,716 participants and isolated non-enteric Gram-negative bacilli in five (2.5%) of 200 with bacteremia. As none of these five isolates was *B. pseudomallei*, there were no confirmed melioidosis cases. Of 323 participants tested by IHA, 142 (44.0%) were male, and the median (range) age was 27 (0–70) years. We identified two (0.6%) cases of probable melioidosis, and 57 (17.7%) were seropositive. The absence of confirmed melioidosis from 9 years of fever surveillance indicates melioidosis was not a major cause of illness.

## INTRODUCTION

The importance of *Burkholderia pseudomallei*, the agent of melioidosis, as a cause of illness in Africa is yet to be fully determined. There have been occasional reports of individual cases and a study indicating that melioidosis is present in coastal Kenya.^1–4^ To our knowledge, there are no published data from Tanzania to indicate whether melioidosis is present,^5^ but prediction models based on environmental suitability and populations at risk suggest that *B. pseudomallei* may be endemic in Tanzania.^6^

*Burkholderia pseudomallei* infection most commonly results in subclinical or self-limiting symptoms in immunocompetent individuals.^7^ Acute symptomatic infection with *B*. *pseudomallei* typically presents with bacteremia, pneumonia, and localized abscesses. *Burkholderia pseudomallei* can be cultured from blood in only a proportion of patients with melioidosis, as bacteremia is thought to occur in 40–60%,^7^ with other patients having localized disease. In diagnostic accuracy studies using latent class models, blood culture has an estimated sensitivity of approximately 60% for melioidosis.^8^ In culture on solid media, *B. pseudomallei* can be overlooked as a contaminant, as colonies appear morphologically similar to common contaminants such as *Pseudomonas stutzeri*.^9^ Finally, *B*. *pseudomallei* can be misidentified as another Gram-negative organism such as *Burkholderia cepacia* or *Chromobacterium violaceum* by routinely used identification systems,^10,11^ and it is not included in standard matrix-assisted laser desorption ionization–time of flight mass spectrometry databases.^12^ As such, melioidosis may be underappreciated or not recognized through routine diagnostic testing in areas where it is uncommon, or the prevalence is unknown.

*Burkholderia pseudomallei* indirect hemagglutination assay (IHA) is a widely used serologic test for melioidosis.^9^ Although use of IHA for the diagnosis of acute melioidosis is limited in endemic countries because of background seropositivity, it remains useful for determining exposure to *B. pseudomallei.*^9^ Population seropositivity in turn informs our understanding of the population-level risk of melioidosis. We sought to understand the prevalence of melioidosis and exposure in northern Tanzania through systematic blood culture testing among patients hospitalized with fever, and IHA serology among a high-risk subset of patients.

## METHODS

### Study setting.

We conducted prospective hospital-based fever surveillance studies at Kilimanjaro Christian Medical Centre (KCMC) and Mawenzi Regional Referral Hospital (MRRH) in Moshi, Tanzania, during the periods of September 17, 2007 through August 31, 2008, September 26, 2011 through May 31, 2014, and September 6, 2016 through May 31, 2019. Kilimanjaro Christian Medical Centre is a 630-bed zonal referral, and MRRH is a 300-bed regional hospital. Moshi (population > 180,000) is the administrative center of the Kilimanjaro region (population > 1.6 million) and is situated at an elevation of approximately 890 m above sea level. The climate is tropical, with rainy seasons from March through May and October through December. Agriculture in northern Tanzania includes smallholder systems involving mixed crop and livestock farming, as well as pastoralism. Continuously irrigated rice farming, an established risk factor for melioidosis, is increasingly practiced by farmers within the area served by our two sentinel site hospitals.^13^

### Study procedures.

We prospectively enrolled pediatric and adult inpatients at KCMC and MRRH during each time period, and during the 2012–2014 time period, we also enrolled outpatients. Adolescents and adults, defined as age ≥ 13 years, were eligible to participate if they had an oral temperature of ≥ 38.0°C, or, during 2012–2014, a history of fever within the previous 72 hours. Infants and children, defined as age ≥ 2 months to < 13 years, were eligible to participate if they had a history of fever in the past 48 hours, an axillary temperature of ≥ 37.5°C, or a rectal temperature of ≥ 38.0°C. We enrolled consecutive eligible inpatients and, during 2012–2014, every second eligible outpatient. Outpatients were included as the primary study sought to describe the incidence of bacterial zoonoses and bloodstream infections, which included diseases that might present to and be managed in the outpatient setting, such as typhoid fever.^14,15^ All patients were enrolled within 24 hours of admission. After obtaining informed consent, a trained study team member completed standardized case report forms and drew blood for culture and serologic testing. The case report form varied during each time period but included demographic details, symptoms, and use of antibacterial drugs before enrollment. Information regarding rainfall in the 30 days preceding enrollment was obtained for the 2012–2014 period from the Tanzanian production company rainfall station near Moshi.

### Blood culture.

We drew blood for a single aerobic blood culture from participants at enrollment, which was loaded into the BacT/ALERT 3D microbial detection system (BioMérieux, Marcy-l’Étoile, France), and incubated for up to 5 days. We inoculated 4 mL of blood into BacT/ALERT pediatric FAN aerobic bottles (2007–2008 and 2012–2014) or pediatric PF plus bottles (2016–2019) (BioMérieux) for pediatric participants (aged < 13 years) and 10 mL of blood into BacT/ALERT standard aerobic bottles (2007–2008 and 2012–2014) or FA plus bottles (2016–2019) (BioMérieux) for adult participants (aged ≥ 13 years). Blood cultures were assessed for volume adequacy by measuring the weight before and after inoculation. Adequate blood volume was defined as ± 20% of the target blood volume. Bloodstream isolates were identified by conventional methods: following testing on the API20NE (BioMérieux) biochemical identification system, non–glucose-fermenting Gram-negative bacilli were further tested by *B. pseudomallei* latex agglutination (Mahidol University, Bankok, Thailand) test.^9,16^

### Serology testing.

During the 2012–2014 study, we collected blood for acute serum at enrollment and convalescent serum 4–6 weeks later. Blood was allowed to clot for between 30 and 60 minutes. It was then centrifuged for 15 minutes at 1,126–1,455 relative centrifugal force to separate serum. Serum was stored at −80°C. Serum specimens were batch shipped on dry ice from Moshi, Tanzania, to Atlanta, GA, for testing. Serology testing was performed at the U.S. CDC. The convalescent serum of a subset of participants considered at high epidemiologic risk of melioidosis on the basis of admission within 30 days of rainfall was tested using *B*. *pseudomallei* IHA. We performed *B. pseudomallei* IHA on paired acute and convalescent sera of participants with a reciprocal titer ≥ 40 on their convalescent serum. We performed *B. pseudomallei* IHA using antigen pooled from two clinical *B*. *pseudomallei* isolates from Southeast Asia and Australia following standard U.S. CDC laboratory protocols.^17^

### Case definitions.

A confirmed case of melioidosis was defined as isolation of *B. pseudomallei* from blood culture, probable melioidosis as a ≥ 4-fold rise in antibody titers between acute and convalescent sera, and seropositivity as a single reciprocal antibody titer ≥ 40.^18,19^

### Statistical analysis.

Case report form and laboratory data were entered using the Cardiff Teleform system (OpenText, Waterloo, Canada) into an Access database (Microsoft Corporation, Redmond, WA). Descriptive analyses, including median and range for continuous variables and proportions for categorical variables, were performed using Stata, version 16.0 (StataCorp, College Station, TX).

### Research ethics.

This study was conducted in accordance with the Declaration of Helsinki. It was approved by the KCMC Research Ethics Committee (#295), the Tanzania National Institute for Medical Research National Ethics Coordinating Committee (NIMR1HQ/R.8cNo1. 11/283), Duke University Medical Center Institutional Review Board (IRB#Pro00016134), and the University of Otago Human Ethics Committee (Health) (H15/055). Written informed consent was obtained from all participants or their guardians.

## RESULTS

We enrolled 3,716 participants who had blood culture performed. Participant characteristics are shown in Table 1. Of note, the median (range) age was 20 (< 1, 84) years. The median (range) duration of illness was 4 (1, 120) days. Of 1,752 participants for whom corresponding rainfall data were available, there were 1,472 (84.0%) who presented within 30 days of rain.

**Table 1:** Characteristics of participants undergoing blood culture, Tanzania, 2007–2019 (*N* = 3,716)

	*N*	*n* (%)
Demographic characteristics		
Age (years), median (IQR)	3,573	20.3 (2.0–39.3)
Gender, male	3,668	1,833 (50.0)
Risk factors		
Rainfall in 30 days before admission (mm), median (IQR)	1,738	24.0 (1.8–67.7)
Farming occupation	1,445	261 (18.1)
Worked in rice field	1,445	23 (1.6)
Self-reported HIV-infected or positive HIV serology	2,052	656 (32.0)
Clinical history		
Illness duration (days), median (IQR)	3,334	4 (3–9)
Cough	3,234	1,936 (59.9)
Dyspnea	3,227	2,216 (31.3)
Headache	2,480	1,551 (62.5)
Myalgia	2,371	947 (40.0)
Rash or cutaneous lesion	2,076	158 (7.6)

IQR = interquartile range; *N* = number of participants with data available.

Among those with data available, 2,658 (73.1%) of 3,637 participants had adequately filled blood culture bottles. Among pediatric participants, 734 (51.4%) of 1,427 blood culture bottles were adequately filled, and among adults 1,924 (87.1%) of 2,210, blood culture bottles were adequately filled. Of responding participants, 849 (32.6%) of 2,607 reported taking antibacterial drugs before enrollment. Non-enteric Gram-negative bacilli were isolated from the blood culture of five (0.1%) participants. None was determined to be *B. pseudomallei*, so no cases of confirmed melioidosis were identified.

Among 323 participants tested by IHA, the median (range) age was 27 (0–70) years, and the median (range) duration of fever was 4 (1–60) days. Other characteristics of participants tested by IHA are shown in Table 2. Forty-four (13.6%) reported being HIV infected. Two (0.6%) cases of probable melioidosis were identified through paired serology, both of whom survived until follow-up. Neither reported recent travel outside Kilimanjaro region. The first case of probable melioidosis was a 6-year-old boy with a 2-day history of fever, rigors, cough, and dyspnea. The participant and his guardians reported a previous negative HIV test. The presence of other immune-suppressing diseases, including diabetes mellitus,^7^ or medications was not ascertained. He reported exposure to standing water around his house and walking barefoot in the mud during the preceding 30 days. A discharge diagnosis of pneumonia was recorded by the treating clinical team. The patient was treated with oral azithromycin. The second case of probable melioidosis was a 36-year-old woman with a 3-day history of fever, rigors, cough, and joint pain. She reported a previous negative HIV test. The presence of other immune-suppressing diseases or medications was not ascertained. She reported no exposure to surface or standing water around her house, and she did not report walking barefoot in the preceding 30 days. She was treated with analgesia and without antimicrobials. A discharge diagnosis of rheumatoid arthritis was recorded by the clinical team.

**Figure 1.**
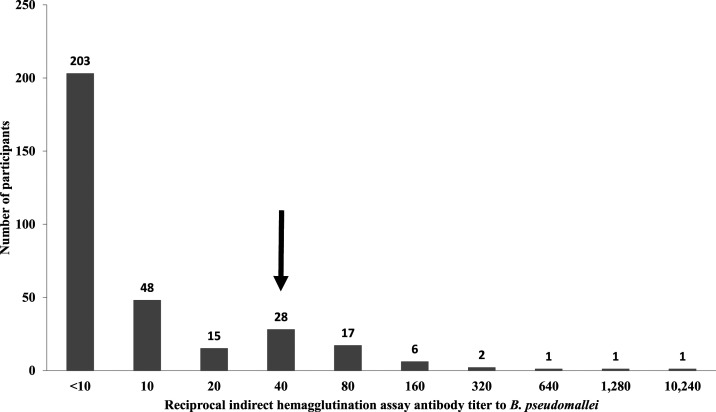
Distribution of *Burkholderia pseudomallei* indirect hemagglutination test reciprocal antibody titers among febrile patients, Tanzania, 2012–2014. Key: Arrow at a reciprocal titer of 40 indicates cutoff for seropositivity.

We identified 57 (17.7%) participants who met the definition for *B. pseudomallei* seropositivity. The highest reciprocal antibody titer was 10,240 (Figure 1). Among participants who were seropositive, 24 (42.1%) were male, and the median (range) age was 23 (< 1, 84) years. Of seropositive participants, 14 (24.6%) reported exposure to surface water compared with 50 (18.8%) seronegative participants (*P* = 0.32), 19 (33.3%) reported the presence of standing water around their house compared with 71 (26.8%) of seronegative participants (*P* = 0.32), and 26 (44.6%) reported walking barefoot in the preceding 30 days compared with 118 (44.4%) of seronegative participants (*P* = 0.86).

## DISCUSSION

This study did not detect a confirmed case of melioidosis in northern Tanzania despite nearly a decade of blood culture in more than 3,700 patients hospitalized with fever. Melioidosis was unlikely to be a major cause of severe febrile illness in northern Tanzania during the study period. Across three study surveillance periods, we cultured blood of consecutive patients hospitalized with fever and systematically investigated isolates to identify *B*. *pseudomallei*. The absence of *B*. *pseudomallei* blood isolates among our patient population does not preclude the occurrence of melioidosis in our region. In Kilifi, Kenya, four *B. pseudomallei* isolates were recovered from > 66,000 blood cultures to provide an estimated annual incidence of 0.2 cases per 100,000 people.^4^ Nonetheless, our findings would indicate that if present melioidosis is likely to be a rare cause of bacteremia in northern Tanzania.

Two participants met the case definition for probable melioidosis. Both cases had negative blood cultures but did not have sputum or other potentially relevant samples cultured. Although each probable case reported a clinical illness that was compatible with melioidosis, the clinical features were nonspecific, and both survived the acute illness without receiving antimicrobials active against *B. pseudomallei*.^7^ Furthermore, we did not ascertain whether either patient had host risk factors for melioidosis such as diabetes or immune suppression. As IHA has limitations in the diagnosis of melioidosis and is not recommended as a diagnostic test in endemic areas,^9^ it is uncertain whether their illness was due to melioidosis.

Almost 20% of participants who underwent IHA testing in our study were seropositive to *B. pseudomallei*. Whether IHA seropositivity indicates exposure to *B. pseudomallei* or an antigenically similar organism such as *Burkholderia mallei* or another *Burkholderia* spp. is uncertain.^20^ To our knowledge, there have not been data published on *B. pseudomallei* seropositivity in Tanzania.^21^ In Uganda, a study by Frazer^22^ identified 25 (5.9%) of 426 individuals as seropositive to *B. pseudomallei* using IHA. The Frazer study surveyed healthy adults from the capital, Kampala, as well as rural areas, and most seropositive participants had reciprocal antibody titers ≤ 160. The relatively high seroprevalence and the absence of bacteremia seen in our study are notable. The seroprevalence in our study was higher than a recent report from Australia,^23^ but lower than a study in Thailand.^24^ Both countries are highly endemic for melioidosis, with annual incidence estimates in northern Australia of up to 50 cases per 100,000 people.^25^ In Thailand, it is considered that 1 in 4,600 antibody-producing exposures result in clinically apparent melioidosis.^24^ A smaller ratio (i.e., fewer exposures per clinically apparent case) is estimated for Australia.^23^ Although exposure to *B. pseudomallei* is a plausible cause of seropositivity, exposure to antigenically similar organisms, such as *B. mallei* and *Burkholderia thailandensis*, is also possible.^20,26^ *Burkholderia mallei*, the agent that causes glanders, serologically cross-reacts with *B. pseudomallei* but is rarely identified in Tanzania.^20,27^ *Burkholderia thailandensis* is a nonpathogenic species that is not known to be present in Tanzania, but has been identified in West Africa.^28^ Further work is needed before it can be determined whether or not melioidosis is endemic in northern Tanzania. Soil sampling using established guidelines to determine whether *B. pseudomallei* or an antigenically similar bacterium is present would be a useful strategy in addition to continued clinical surveillance.^29^

Our study determined that many participants had exposure to surface water and undertook activities that might place them at risk for melioidosis. However, we had selected participants for testing if they had presented within 30 days of rainfall, as recent rainfall is an established risk factor for melioidosis in endemic countries. The strategy of testing only participants with known risk factors introduced bias, which precluded a formal risk factor analysis.

Although our study used a systematic approach to evaluating blood culture isolates, limitations in the methodology may underestimate the prevalence of melioidosis. In particular, not all patients with fever were enrolled; some participants had inadequately filled blood culture bottles and some participants reported having taken antibacterial drugs before enrollment.^30^ In addition, culture solely of blood for *B. pseudomallei* will have underestimated the prevalence, and systematic culture of urine, sputum, pus and throat swabs is likely to have increased diagnosis. The *B. pseudomallei* seroprevalence cannot be considered a true community prevalence because testing was targeted toward a subset of patients.

In conclusion, we have not identified culture-confirmed melioidosis despite nearly a decade of careful surveillance. Based on these data, we would not recommend that clinicians in northern Tanzania treat febrile patients with antimicrobials that cover melioidosis empirically. However, given the IHA results, it remains possible that melioidosis is present in northern Tanzania and an index of suspicion of melioidosis among patients with fever, and culture of blood and multiple clinical specimen types remain critical to ensure that persons with melioidosis are appropriately identified and treated.

**Table 2:** Characteristics of participants undergoing *Burkholderia pseudomallei* indirect hemagglutination testing, Tanzania, 2012–2014 (*N* = 323)

	*n* (%)
Demographic characteristics	
Age (years), median (IQR)	27 (5–40)
Gender, male	142 (44.0)
Risk factors	
Rainfall in 30 days before admission (mm), median (IQR)	46 (27–72)
Farming occupation	66 (20.4)
Self-reported HIV-infected	44 (13.6)
Clinical history	
Fever duration (days), median (IQR)	4 (2–7)
Cough	200 (61.9)
Dyspnea	101 (31.3)
Headache	217 (67.2)
Myalgia	140 (43.3)
Rash or cutaneous lesion	24 (7.4)

IQR = interquartile range.
